# Age-Dependent Dynamics of Maternally Derived Antibodies (MDAs) and Understanding MDA-Mediated Immune Tolerance in Foot-and-Mouth Disease-Vaccinated Pigs

**DOI:** 10.3390/vaccines10050677

**Published:** 2022-04-24

**Authors:** Sehee Shin, So Hui Park, Jong-Hyeon Park, Su-Mi Kim, Min Ja Lee

**Affiliations:** Animal and Plant Quarantine Agency, 177 Hyeoksin 8-ro, Gimcheon-si 39660, Gyeongsangbuk-do, Korea; ssh9147@korea.kr (S.S.); sohui33@korea.kr (S.H.P.); parkjhvet@korea.kr (J.-H.P.); beliefsk@korea.kr (S.-M.K.)

**Keywords:** foot-and-mouth disease, maternally derived antibodies, interference, characteristics, regulatory T cells

## Abstract

Vaccine-induced active immunity in young animals may be compromised via interference caused by maternally derived antibodies (MDAs). Since the level, titer, and half-life of MDAs vary per individual, it is difficult to determine the appropriate timing of foot-and-mouth disease (FMD) vaccination in the field. In order to better understand the age-dependent characteristics of MDA in sows and piglets as well as the phenomenon of reduced vaccine-mediated active immunity due to MDAs, this study sought to determine antibody titers through structural protein (SP) O, A ELISA analyses, and virus-neutralizing (VN) antibody titers as well as their half-lives in the sera of sows and piglets derived from FMD-vaccinated mother. Furthermore, immunoglobulin (Ig) subtypes, such as IgG, IgM, and IgA, in serum were also evaluated. To understand the correlation between the inhibition of vaccine-mediated active immunity by MDA-mediated passive immunity and regulatory T (T_reg_) cells, T_reg_-related cytokine levels were explored. Our findings will help to predict the optimal timing of vaccination for overcoming MDAs and inducing a robust vaccine-mediated immune response in young individuals vaccinated against FMD. They also add to our understanding of MDA characteristics and interference, providing insight for the development of innovative strategies and novel FMD vaccine for overcoming such interference.

## 1. Introduction

Vaccination is a major approach for disease control in countries with an outbreak of foot-and-mouth disease (FMD, for the abbreviation list see Table S1). The commercially available FMD vaccine induces antibodies with a short half-life, requiring regular and repetitive vaccination in both cattle and pigs. Vaccination-induced antibodies in the mother are delivered to calves or piglets as maternally derived antibodies (MDAs) through the ingestion of placenta or colostrum for the formation of passive immunity. Pathogen-specific antibodies and other immune mediators are transmitted from mother to fetus during pregnancy via the placenta and then by breast milk-derived immunity after birth. Antibody-mediated passive immunity can prevent the newborn from infections, and neonatal immunity depends on the maternal concentration of respective specific antibodies during pregnancy [1]. The maternal immune system during fetal development is known to be very active and dynamic. It interacts with fetal immune cells to create a prenatal environment that supports pregnancy but also affects the programming of the fetal immune system. The placenta provides a barrier that can control and impede the transfer of harmful substances from the mother to the fetus. Therefore, a specific and active transport mechanism is required to deliver the maternal pathogen-specific antibody (MDA) to the fetus. During pregnancy, IgG antibodies (passive humoral immunity) are transferred from a mother to a fetus in a highly controlled manner mediated by the neonatal Fc receptor (FcRn) for IgG. It is expressed in the placental syncytial trophoblast, which characteristically binds to the Fc fragment of an IgG antibody and promotes transport to parts of the body in need of specific immunity. Although the transmission of Ig via the placenta to the fetus is small, significant levels are transmitted at the end of the pregnancy. The majority of passively acquired Ig is derived from milk after birth. All Ig acquired in the uterus and later across the intestinal barrier is exclusively of the IgG isotype, although milk has a large preponderance of IgA [2,3].

MDAs protect calves and piglets against initial FMD virus (FMDV) infection, yet protection does not last long. When the FMD vaccine is injected early in young animals, potential active immunity becomes subject to interference by passive immunity, which compromises vaccine efficacy [4]. Therefore, the current FMD vaccination program in Korea recommends that calves, piglets (usually between 8 and 12 weeks of age, depending on farm conditions), young goats, and deer be administered after 2 months of age, when the MDA level has decreased, and the second dose should be administered 4 weeks later.

Since the level, titer, and half-life of MDAs differ between individuals, it is difficult to determine the optimal timing of vaccination against FMD in the field.

Thus far, various studies have explored MDA interference and the compromised efficacy of the FMD vaccine. Elnekave et al. found that increasing the frequency of vaccination in calves over 3 months of age helps antibody generation [5]. Kim et al. reported that a satisfactory increase in titer was not observed even in the secondary vaccination group due to MDA interference, which called for the improvement of the commercially available vaccine [6]. In addition, research results have suggested that booster shots after inoculation with a vaccine with a potency of 6PD_50_ (50% protective dose) or higher would help overcome MDAs [7]. Further, the evaluation and goal setting of vaccine coverage, herd immunity, and vaccine field efficacy would be required [8]. Researchers have also explored the optimal timing of vaccination in light of MDA reduction, with one study demonstrating that vaccination in late pregnancy increases the duration of MDAs and IgG levels in the colostrum [9]. It was also argued that continuous monitoring is required, as the MDA level of piglets is highly dependent on the antibody titer of sows, meaning that the inoculation timing of piglets may vary depending on the vaccination program and the quality of the vaccine given to sows [10]. While considerable research efforts have focused on the interference caused by MDAs, studies suggesting a specific method for effectively overcoming MDAs and achieving a strong vaccine-induced immune response in young individuals are scarce. Despite active research into vaccine development for overcoming MDAs in birds or pigs, including Newcastle disease virus [11,12], avian influenza virus [12,13], porcine reproductive and respiratory syndrome virus [14], porcine circovirus-2 [15,16], influenza A virus [17], and classical swine fever [18] vaccines, in addition FMD vaccines, few systematic studies have performed MDA characterization and employed immunological approaches.

The relationship between regulatory T cells (T_regs_) and immune tolerance and the role of T_regs_ in enhancing maternal immune tolerance during pregnancy have been reported [19,20]. T_regs_ play a major role in maintaining immune homeostasis, preventing autoimmunity, regulating inflammation, minimizing collateral damage to tissues as well as maintaining maternal–fetal immune tolerance [19,21]. T_regs_ suppress the proliferation of naive T cells into effector T cells (T_eff_). Recent report also supported that T_regs_ block maternal T_eff_ and that maternal IgG and IgA antibodies dampen mucosal T helper cell responses in early life [22]. They can also inhibit the effector activity of differentiated CD4^+^ and CD8^+^ T cells and the function of natural killer (NK) cells, natural killer T (NK T) cells, B cells, macrophages, osteoclasts, and dendritic cells (DCs). T_regs_ inhibit the proliferation of responder T cells and cytokine such as IL-2 production when stimulated by antigen in the presence of antigen presenting cells (APCs). Several mechanisms of T_reg_-mediated inhibition have been proposed, including immunosuppressive cytokine secretion by T_regs_, cell-contact-dependent inhibition, and the functional modification or death of APCs. T_regs_ and immunosuppressive cytokines such as IL-10 and TGFβ result in a state of immune tolerance by inducing an “immune regulatory network” [23].

In order to overcome the interference of MDAs, which represents a major limiting factor for commercially available FMD vaccines, this study aimed to elucidate the age-dependent properties of MDAs in sows and piglets as well as to evaluate MDA interference in relation to antibody titers, virus-neutralizing (VN) titers, and their half-lives. We also sought to elucidate whether MDA interference is associated with T_reg_-induced immune tolerance. These data would allow for the prediction of optimal vaccination timing for overcoming MDA-associated interference. Furthermore, a systematic understanding of MDA interference can be applied to vaccine development studies that can elicit a robust immune response by overcoming MDA interference when vaccinating young individuals.

## 2. Materials and Methods

### 2.1. Sampling

To investigate the properties MDAs, five sows and 10 piglets from each sow were screened. The vaccination history of sows used in present study was as follows. Sows were vaccinated periodically after 2 months of age (usually 8–12 weeks of age) with the commercial vaccine (Bioaftogen^®^, FMD O1 Campos, A 2001 Argentina and A24 Cruzeiro) from Biogénesis Bagó (Buenos Aires, Argentina), which was emulsified with the adjuvant (Montanide ISA 50, 80%/20%, *v*/*v*, water in oil) according to the vaccination program of Korea. Sows were not vaccinated for 4 weeks before and after fertilization and were vaccinated around 60–80 days during a gestation period of about 115 days. We used sows of the same age, raised on the same outdoor farm, and the animals were vaccinated with the same dose of the same commercial vaccine at the same time. 

The sows were screened based on antibody titers (percent inhibition (PI) value: >50% for PrioCheck^TM^ kit; >40% for VDPro^®^ kit, antibody-positive (+)) via ELISA for SP O and SP A, respectively, and VN titers (>1.65 Log_10_, 45-fold, protective level).

For sows (n = 5), blood was collected 7 days before farrowing. For piglets (n = 10/sow, total n = 50) born to sows, blood was collected on 7, 14, 28, 42, 56, 84, and 112 days post-birth (dpb). Serum samples were stored at −80 °C until tests were performed. The animals were isolated in closed containments (Animal Biosafety Level 3, ABSL-3) at the Animal and Plant Quarantine Agency during the study. After arrival at our ABSL facility, all animals were kept in cages with *ad libitum* access to food and water. Animals were allowed at least a week of adaptation before experiments. They were kept under a 12/12-h light/dark cycle, a temperature of about 22 °C, and relative air humidity of about 50%. All animal experiments were performed according to institutional guidelines, with approval from the Ethics Committee of the Animal and Plant Quarantine Agency (accreditation number: IACUC-2021-584).

### 2.2. ELISA for the Detection of Structural Protein (SP) Antibodies

To detect SP antibodies in sera, PrioCheck^TM^ FMDV type O or FMDV type A (Prionics AG, Schlieren, Switzerland) and VDPro^®^ FMDV type O or FMDV type A (Median Diagnostics, Chuncheon, Gangwon-do, Korea) were used. We performed both SP O ELISA and SP A ELISA because sows had both FMDV type O- and FMDV type A-specific antibodies. Since the type of FMDV antigen coated on the ProCheck^TM^ kit and the VDPro^®^ kit is different, the antibody titer appears differently depending on the structural protein of the coated antigen in plate. Therefore, we measured the antibody titer using both types of kit to prevent the antibody titer from being underestimated by the characteristics of the antigen coated on the kit.

Absorbance in the ELISA plate was converted to PI value. When the PI value was 50% for the PrioCheck^TM^ FMDV kit or 40% and above for the VDPro^®^ FMDV kit, animals were considered antibody positive [16,17,18].

### 2.3. Virus Neutralization Test (VNT)

A VNT was performed based on the guidelines set forth by the World Organization for Animal Health (OIE) manual [24], as described by Lee et al. and Swaney [25,26,27,28]. Briefly, sera were heat-inactivated at 56 °C for 30 min in a water bath. LFBK (bovine kidney) cell density was adjusted to form a 70% monolayer, and 2-fold serial dilutions of serum samples (1:8–1:1024) were prepared. The diluted serum samples were then incubated with a 100-tissue culture infectious dose (TCID)_50_/0.5 mL homologous virus for 1 h at 37 °C. After 1 h, LFBK cell suspension was added to all wells. After 2–3 days, CPE was evaluated to determine the titers, which were calculated as Log_10_ of the reciprocal antibody dilution required to neutralize 100 TCID_50_ of the virus [29,30]. FMDV O1 Campos, FMDV A2001 Argentina, and FMDV A24 Cruzeiro were used for VNT.

### 2.4. Isotype-Specific Antibody Immunoassays

To detect specific antibody isotypes, ELISA for porcine IgG, IgA, and IgM (Bethyl Laboratories. Inc., Montgomery, TX, USA) were performed on sera according to the manufacturer’s instructions. Briefly, 100 μL/well of serially diluted sera and standards were added to the appropriate wells, and the plates were incubated at RT for 1 h. After another washing and drying step, 100 μL/well of the biotinylated detection antibodies were added to all wells, and the plates were incubated at RT for 1 h. The wells were washed and patted dry, 100 μL/well of diluted streptavidin–horseradish peroxidase conjugate was added, and the plates were incubated at RT for 30 min. Subsequently, the plates were washed again and dried. The peroxidase reaction was developed with 100 μL/well of 1× TMB solution for 30 min at RT and stopped with 100 μL 2 N H_2_PO_4_. Absorbance was measured within 30 min using a Hidex 300SL spectrophotometer (Hidex, Turku, Finland) set at 450 nm [16,17,18].

### 2.5. Cytokine ELISA

ELISAs for porcine IL-2, IL-10, and TGFβ (Cloud-Clone Corporation, Houston, TX, USA) in serum were performed according to the manufacturer’s instructions. The subsequent steps were as described for antibody isotype-specific immunoassays [25,26,27].

### 2.6. Statistics

All quantitative data are expressed as the mean ± standard error (SEM) unless otherwise stated. The statistical significance of differences between groups was assessed using two-way ANOVA followed by Bonferroni post hoc test or one-way ANOVA followed by Tukey’s post hoc test. * p < 0.05; ** *p* < 0.01; *** p < 0.001; and **** *p* < 0.0001. Parametric tests were also used to compare groups. Survival curves were built via the Kaplan–Meier method, and differences were analyzed using the log-rank sum test. GraphPad Prism 9 (GraphPad, San Diego, CA, USA) software and IBM SPSS software (IBM Corp., Armonk, NY, USA) were used for all statistical analyses.

## 3. Results

### 3.1. Age-Dependent Dynamics of Antibody Titers in Sows and Piglets

**Figure 1:**
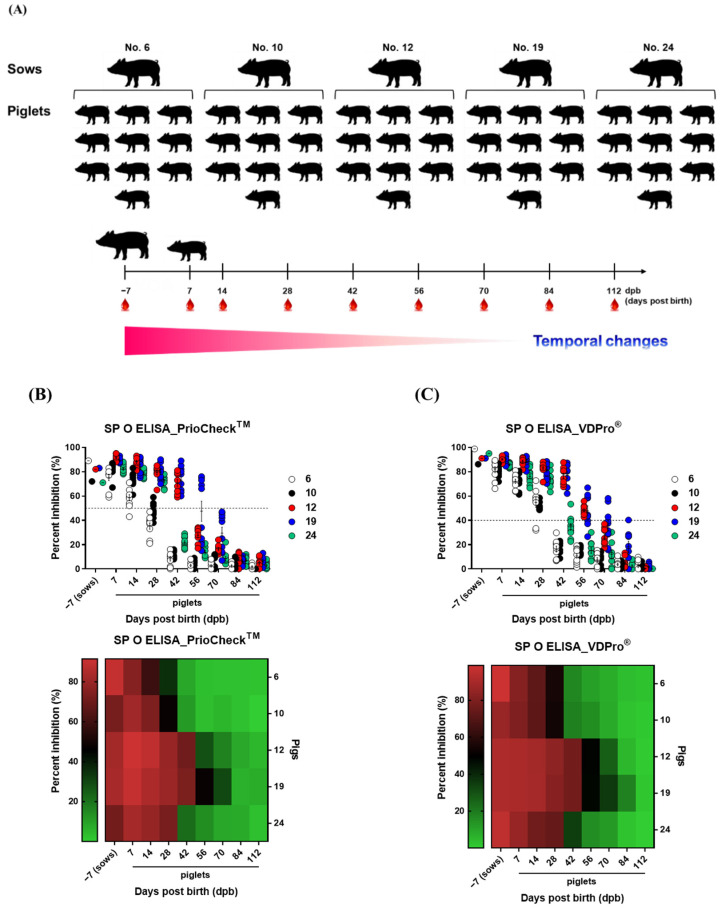

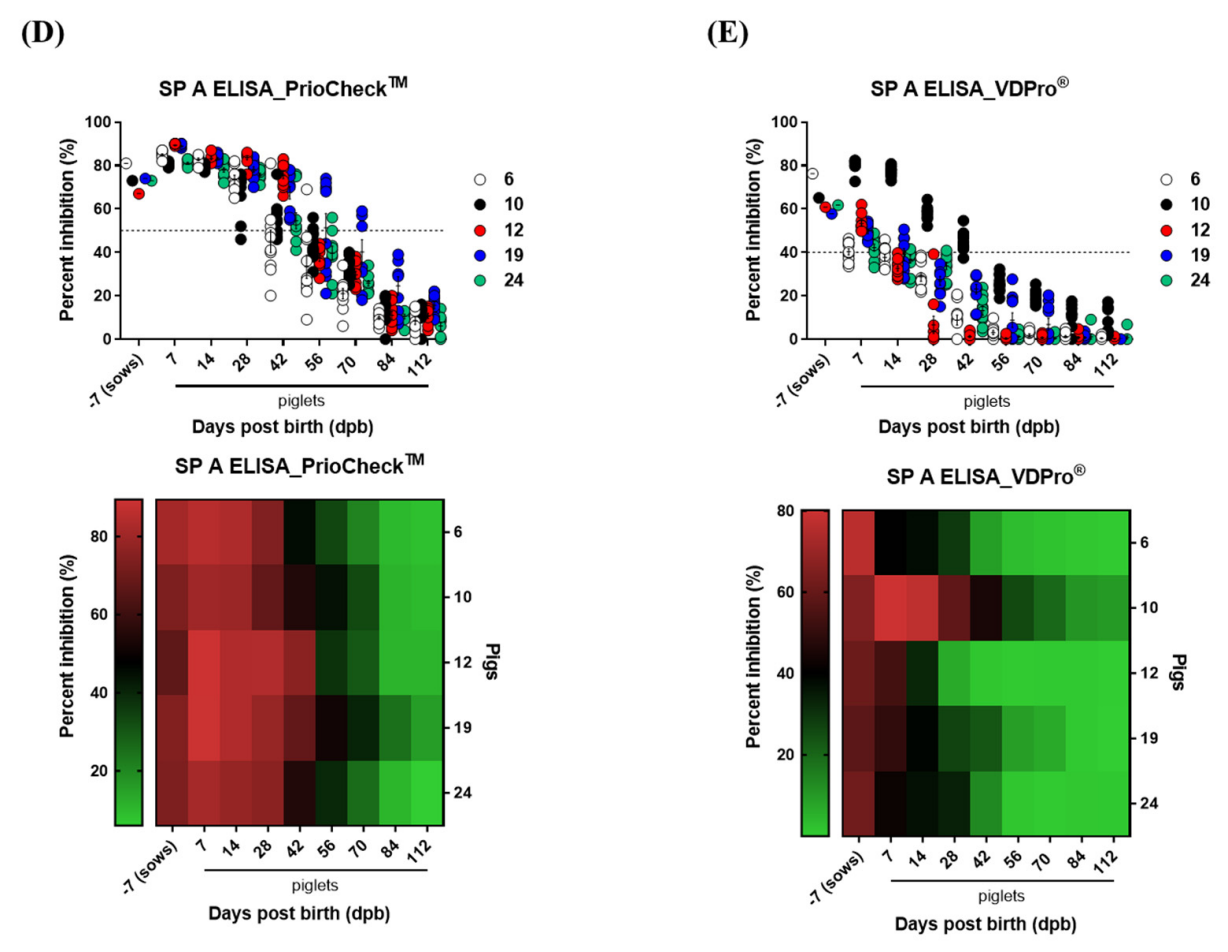
Age-dependent dynamics of maternally derived antibodies (MDA) in the sera of sows and piglets determined via SP O, A ELISA. To understand the properties of MDAs, five sows and 10 piglets from each sow were screened. For sows, blood was collected 7 days before farrowing. For the piglets born to sows, blood was collected on 7, 14, 28, 42, 56, 84, and 112 days post-birth. Serum antibody titer was determined via SP O, A ELISA using the PrioCheck^TM^ kit and VDPro^®^ kit. Data are presented as the mean ± SEM of triplicate measurements (*n* = 5 for sows and 10 piglets/sow). Data are shown in scatter plots and heat maps. The black dotted line represents the threshold of antibody positive (+) or negative (−). (**A**–**E**) represent: (**A**) Strategy for this study; (**B**) SP O ELISA determined using the PrioCheck^TM^ kit; (**C**) SP O ELISA determined using the VDPro^®^ kit; (**D**) SP A ELISA determined using the PrioCheck^TM^ kit; (**E**) SP A ELISA determined using the VDPro^®^ kit.

In order to determine the antibody titers (PI, %) and half-life in pigs vaccinated against FMD, periodic blood samples from pregnant sows (n = 5) and their piglets (n = 10 per sow) were collected (sows: 7 days before farrowing, piglets: 7, 14, 28, 42, 56, 70, 84, and 112 days after birth), and age-dependent antibody titer dynamics were described (Figure 1). SP O and SP A antibody titers were measured using both the PrioCheck^TM^ kit and the VDPro^®^ kit. Antibody titers were determined via SP O ELISA using the PrioCheck kit. Various piglets between 28 and 56 dpb still showed positive titers, and after 70 dpb, all piglets were antibody negative. Titers determined via SP O ELISA using the VDPro^®^ kit showed large variation at 42 to 70 dpb, and the time point at which all tested subjects were negative was 84 dpb. Unlike changes in antibody titers determined via SP O ELISA, SP A ELISA using the PrioCheck^TM^ kit indicated a gradual decrease, with large variation at 42–70 dpb, and all subjects were antibody negative after 84 dpb. Antibody titers determined by SP A ELISA using the VDPro^®^ kit decreased much faster. After a sharp decrease from 7 to 42 dpb, all subjects were antibody negative at 56 dpb (Figure 1A–E).

### 3.2. Age-Dependent Dynamics of VN Titers

**Figure 2:**
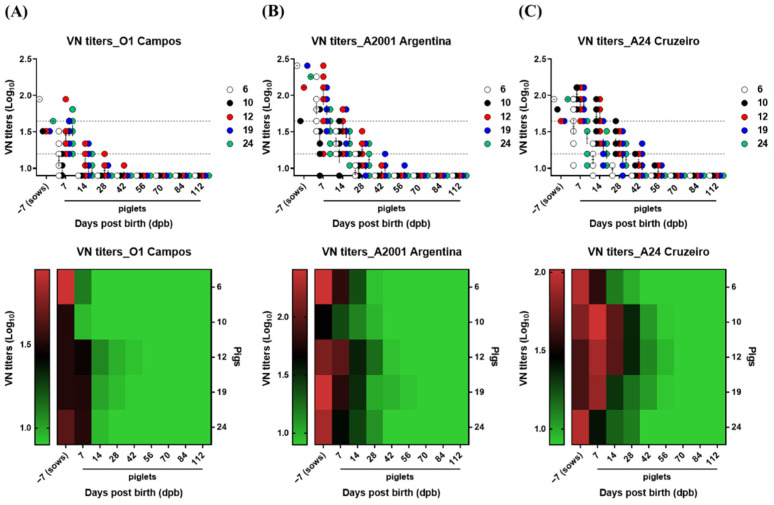
Age-dependent dynamics of maternally derived VN titers for O1 Campos, A2001 Argentina, and A24 Cruzeiro in the serum of sows and piglets. To understand the properties of maternally derived antibodies (MDAs), five sows and 10 piglets from each sow were screened. For sows, blood was collected 7 days before farrowing. For piglets born to sows, blood was collected on 7, 14, 28, 42, 56, 84, and 112 days post-birth. The virus-neutralizing antibody (VN) titer for O1 Campos, A2001 Argentina, and A24 Cruzeiro was measured in sampled serum. The black dotted line represents VN titers of 1.65 Log_10_ or 1.2 Log_10_. Data are presented as the mean ± SEM of triplicate measurements (*n* = 5 for sows and 10 piglets/sow). Figures were presented as scatter plots and heat maps. (**A**–**C**) represent: (**A**) VN titers for O1 Campos; (**B**) VN titers for A2001 Argentina; (**C**) VN titers for A24 Cruzerio.

In order to investigate the age-dependent dynamics and half-life of VN titers based on MDAs in pigs vaccinated against FMD, blood from sows (*n* = 5) and the piglets of each sow (*n* = 10) was collected periodically as previously described. The vaccination records of the sows used in this experiment indicated that the commercial trivalent vaccine (FMDV strain O1 Campos, A 2001 Argentina and A24 Cruzeiro) from Biogénesis Bagó (Buenos Aires, Argentina) had been used. Thus, VN titers were measured using the O1 Campos, A2001 Argentina, and A24 Cruzeiro strains. The VN titers of sows were highest for A2001 Argentina, followed by A24 Cruzeiro and O1 Campos. The VN titers for A2001 Argentina and A24 Cruzeiro reached below the defensive line of 1.65 (Log_10_) at 28 dpb for most piglets. However, the decrease in VN titers below 1.2 (Log_10_) showed a more gradual decrease for A24 Cruzeiro than for A2001 Argentina (Figure 2A–C).

### 3.3. MDA Half-Life

**Figure 3:**
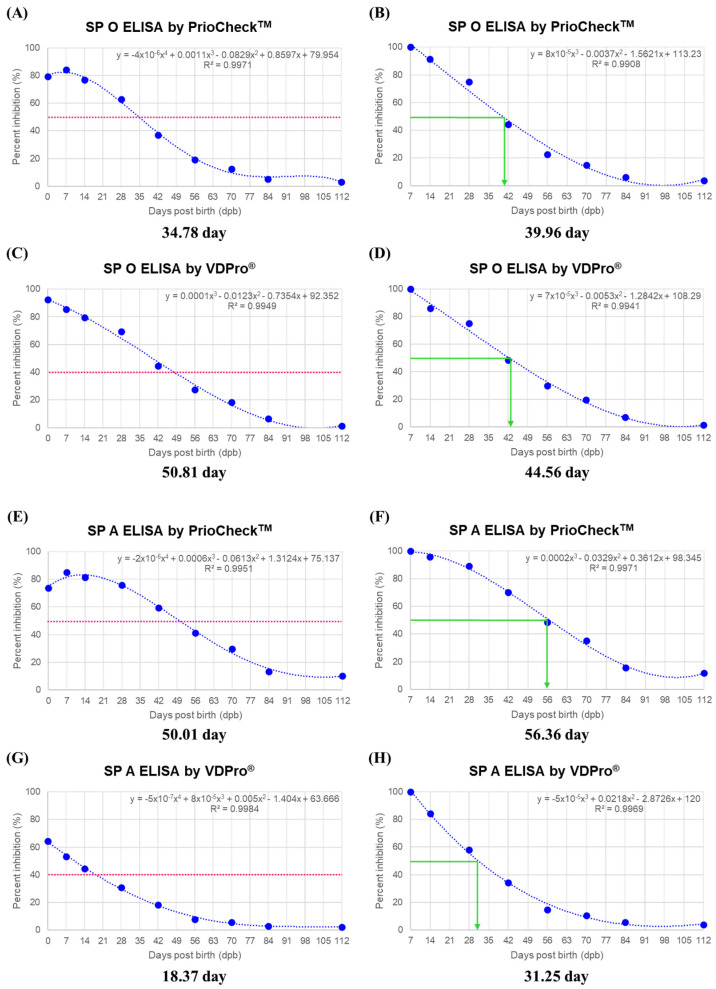
Time kinetics and half-life of maternally derived antibodies (MDA) as measured via SP O, A ELISA. To understand the properties of MDAs, five sows and 10 piglets from each sow were screened. For sows, blood was collected 7 days before farrowing. For piglets born to sows, blood was collected on 7, 14, 28, 42, 56, 84, and 112 days post-birth. Antibody titer in the sampled serum was measured via SP O, A ELISA using PrioCheck^TM^ and VDPro^®^ kits. Data are presented in regression curves for time kinetics and half-life of MDAs. The red dotted line and the green solid line represent the threshold of antibody positive (+) or negative (−), and the half-life of MDAs, respectively. (**A**–**H**) represent: (**A**,**B**) Time kinetics and half-life of MDAs determined via SP O ELISA using the PrioCheck^TM^ kit; (**C**,**D**) time kinetics and half-life of MDAs determined via SP O ELISA using the VDPro^®^ kit; (**E**,**F**) time kinetics and half-life of MDAs determined via SP A ELISA using the PrioCheck^TM^ kit; (**G**,**H**) time kinetics and half-life of MDAs determined via SP A ELISA using the VDPro^®^ kit. The data represent the mean ± SEM of triplicate measurements (n = 5 for sows or 10 piglets/sow).

Based on the results in Figure 1 and Figure 2, regression curves and equations were derived to determine the half-life of MDAs (Figure 3). We related the PI with the dpb, using as a starting point the PI of sows (considered as time 0) on the left and the PI of the piglets at 7 dpb on the right. Then, in order to estimate the half-life of MDAs, we transformed all the PIs of piglets at the various sampling times, considering the PIs of the first piglet sampling (7 dpb) as 100%. The left panel represents the time kinetics of antibody titer transition from antibody positive to antibody negative, and the right panel represents the half-life of MDAs. As for SP O ELISA using the PrioCheck^TM^ kit, the time for the PI value to reach 50% in the antibody titers was 34.78 days, and with the initial antibody of piglets converted to 100%, the time for the PI value to reach 50%, the half-life, was 39.96 days (Figure 3A). For SP O ELISA using the VDPro^®^ kit, the time for the PI value to reach 40% of antibody titers was 50.81 days, and with the initial antibody of piglets converted to 100%, the half-life of MDAs was 44.56 days (Figure 3B). As for SP A ELISA using the PrioCheck^TM^ kit, the PI value reached 50% at 50.01 days based on raw data, and the half-life of MDAs in piglets was 56.36 days (Figure 3C). For SP A ELISA antibody titer using the VDPro^®^ kit, the PI value reached 40% at 18.37 days based on raw data, and the half-life of MDAa in piglets was 31.25 days (Figure 3D). As for SP A ELISA, the level of MDA half-life determined via the VDPro^®^ kit was significantly different from other values related to MDA half-life.

**Figure 4:**
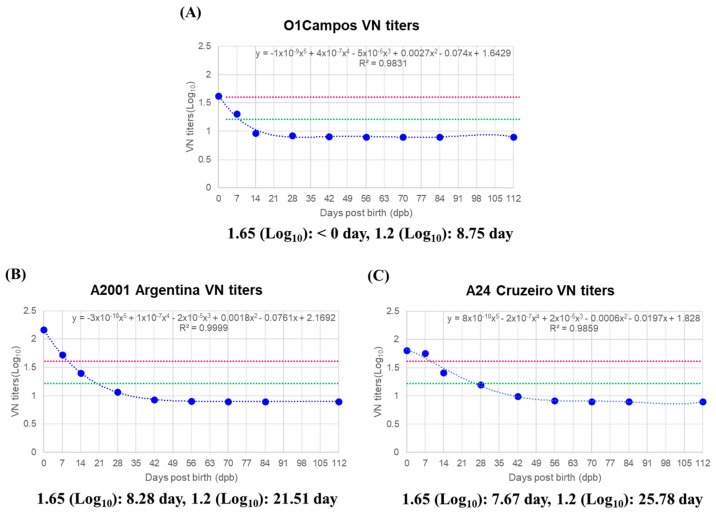
Time kinetics of maternally derived antibodies determined based on VN titers. To understand the properties of maternally derived antibodies, five sows and 10 piglets from each sow were screened. For sows, blood was collected 7 days before farrowing. For piglets born to sows, blood was collected on 7, 14, 28, 42, 56, 84, and 112 days post-birth. Virus-neutralizing antibody (VN) titers for O1 Campos, A2001 Argentina, and A24 Cruzeiro were measured in the sampled serum. Data are presented in regression curves for time kinetics and half-life of MDAs. The red dotted line and the green dotted line correspond to VN titers 1.65 (Log_10_) and 1.2 (Log_10_), respectively. (**A**–**C**) represent: (**A**) Time kinetics of MDA based on VN titers for O1 Campos; (**B**) time kinetics of MDAs based on VN titers for A2001 Argentina; (**C**) time kinetics of MDAs based on VN titers for A24 Cruzeiro. The data represent the mean ± SEM of triplicate measurements (n = 5 for sows or 10 piglets/sow).

The VN titers decreased very fast for O1 Campos, taking <0 days to reach 1.65 (Log_10_) and 8.75 days to reach 1.2 (Log_10_) (Figure 4A). For A2001 Argentina and A24 Cruzeiro, it took 8.28 and 7.67 days, respectively, to reach 1.65 (Log_10_), as well as 21.51 and 25.78 days, respectively, to reach 1.2 (Log_10_), decreasing much faster than the antibody titers determined via SP O, A ELISA (Figure 4B,C).

### 3.4. Kinetics of Total IgG, IgM, and IgA Concentrations in Sows and Piglets

**Figure 5:**
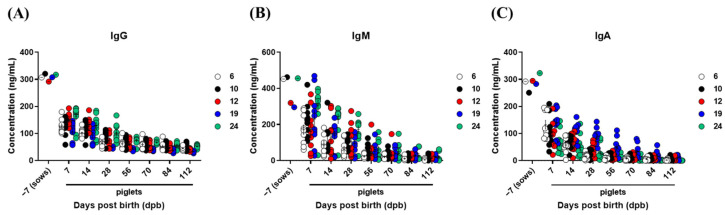
Age-dependent dynamics of IgG, IgM, and IgA in serum from sows and piglets. Five sows and 10 piglets were screened from each sow. For sows, blood was collected 7 days before farrowing, and for piglets born to sows, blood was collected on 7, 14, 28, 42, 56, 84, and 112 days post-birth. The concentrations of IgG, IgM, and IgA were measured using commercially available ELISA kits. Data are present as the mean ± SEM of triplicate measurements (n = 5 for sows or 10 piglets/sow). (**A**–**C**) represent: (**A**) IgG concentration; (**B**) IgM concentration; (**C**) IgA concentration.

As total immunoglobulins induced by vaccines for prophylaxis of other animal diseases may also affect the FMD vaccine-induced immune response in piglets, we determined the kinetics of IgG, IgM, and IgA concentrations after birth (Figure 5). IgG levels (Figure 5A), an indicator of neutralizing antibody titers, IgM (Figure 5B), the first isotype produced following pathogen infection or vaccination, and IgA (Figure 5C), which plays an important role in mucosal immunity, decreased sharply at 7 dpb and showed a tendency of steadily decrease up to 112 dpb. This change in immunoglobulin isotype was similar to the pattern of the decrease in antibody levels determined via SP O ELISA and SP A ELISA (Figure 5A–C).

### 3.5. The Inhibition of Vaccine-Mediated Active Immunity by MDAs and T_reg_ Cells

**Figure 6:**
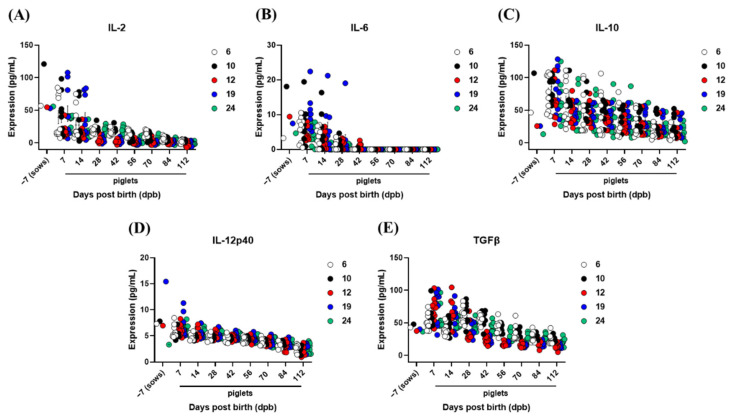
T_reg_-associated cytokine levels in serum from sows and piglets. Five sows and 10 piglets were screened from each sow. For sows, blood was collected 7 days before farrowing. For the piglets born to sows, blood was collected on 7, 14, 28, 42, 56, 84, and 112 days post birth. The concentration of cytokines, including IL-2, IL-6, IL-10, IL-12p40, and TGFβ, was measured using commercially available ELISA kits. Data are presented as the mean ± SEM of triplicate measurements (n = 5 for sows or 10 piglets/sow). (**A**–**E**) represent: (**A**) IL-2 concentration; (**B**) IL-6 concentration; (**C**) IL-10 concentration; (**D**) IL-12p40 concentration; (**E**) TGFβ concentration.

In order to investigate the correlation between MDA-induced interference and T_reg_ in pigs vaccinated against FMD, changes in the expression of T_reg_-related cytokines, such as IL-2, IL-10, and TGFβ were detected. We also compared the levels of Th2 cytokines such as IL-6 as well as cytokines involved in innate and adaptive immunity through the stimulation of APCs such as IL-12 (Figure 6). T_reg_-related cytokine changes exhibited a pattern of decrease similar to the dynamics of antibody titers determined via SP O ELISA and SP A ELISA, with high levels up to 7 dpb in both sows and piglets, followed by a gradual decrease. While IL-2, IL-10, and TGFβ were highly correlated with MDA changes, IL-6 and IL-12 showed a decrease pattern similar to that of MDAs. However, the absolute expression level was lower than that of IL-2, IL-10, and TGFβ, indicating a weak correlation (Figure 6A–E).

## 4. Discussion

FMD is recognized by the OIE as a major infectious disease affecting livestock, causing severe economic losses and reduced productivity due to its rapid spread in both adults and young individuals. In particular, young individuals show a high morbimortality due to myocarditis [24]. The commercial FMD vaccine takes a long time to induce sufficient protective antibody titers (humoral immunity), complicating early defense against the viral infection. Furthermore, the humoral immune response is weaker in some individuals and is often limited by the phenomenon of MDA interference, which compromises the formation of vaccination-induced active immunity. Therefore, there is a pressing need to improve FMD prophylaxis.

Compared to other animals, pigs exhibit large inter-individual differences in the induction of antibody titers and VN titers after vaccination, in addition to an overall weaker response being induced than in cattle. These seem to be due to (1) changes in innate and cellular immune responses in the host due to the expression of proinflammatory and anti-inflammatory cytokines caused by environmental factors in animal breeding farms and (2) interference by passive immunity, such as MDAs, leading to the suppression of antigen-specific antibody production from plasma cells and memory B cells, which contributes to immunological tolerance [31].

To date, studies have reported vaccine-related MDAs for various animal diseases. For example, previous studies characterized MDAs and the transmission mechanism for rabbit hemorrhagic disease virus-2 [32], explored the effect of MDAs on swine influenza A virus epidemiology [33], and explored the effects of MDAs on both humoral and cellular immune responses after vaccination in piglets [34]. However, there are few systematic studies focused on overcoming MDA interference, which requires an improved fundamental understanding of age-dependent changes in MDAs following FMD vaccination, the induction of passive immunity, and immunological tolerance mechanisms.

In order to understand age-dependent MDA characteristics in sows and their piglets, changes in serum antibody and VN titers were determined via SP O and A ELISA in serum (Figure 1 and Figure 2). Antibody titers determined via SP O ELISA using the VDPro^®^ kit and SP A ELISA using the PrioCheck^TM^ kit exhibited similar patterns of decrease (Figure 1). Meanwhile, as O1 Campos, A2001 Argentina, and A24 Cruzeiro strains were used as vaccine strains for the vaccine from Biogénesis Bagó administered to sows, VN titers were measured using homologous virus strains. VN titers in sows were the highest for A2001 Argentina, followed by A24 Cruzeiro > O1 Campos. Furthermore, VN titers for A2001 Argentina in piglets decreased much faster than VN titers for A24 Cruzeiro during the first days after birth (7 to 28 dpb), with both A2001 Argentina and A24 Cruzeiro indicating similar levels of VN titers at 42 dpb (Figure 2).

We then determined the MDA half-life for antibody titers and VN titers (Figure 3 and Figure 4). In order to induce humoral immunity response through vaccination while avoiding interference by MDAs, the first dose was administered to piglets at 8 to 12 weeks of age. Our findings suggest that it takes about 50 days for MDAs to decrease from positive to negative in piglets, based on the antibody titer of sows vaccinated with commercially available vaccines in South Korea (SP O ELISA by VDPro^®^ kit: 50.81 day; SP A ELISA by PrioCheck^TM^ kit: 50.01 day). The half-life of MDAs was 39.96 to 44.56 days (5.71 to 6.37 weeks) for antibody titers determined via SP O ELISA with the PrioCheck^TM^ kit and VDPro^®^ kit, and 56.36 days (8.05 weeks) for titers determined via SP A ELISA with the PrioCheck^TM^ kit. The MDA level in piglets may vary depending on the individual, in addition to differences depending on the kit used to measure antibody titers and the antigen contained in the vaccine.

According to the OIE guideline, a VN titer value below 1.05 (Log_10_) is considered negative, while a value above 1.65 (Log_10_) is considered positive [35]. Efficacy evaluation of FMD vaccine is centered on cattle rather than pigs, and the evaluation criteria are also set based on cattle. In cattle, 1.65 Log_10_ (45-fold neutralizing antibody titer) and 1.2 Log_10_ (16-fold neutralizing antibody titer) represent over 90% and 50% protection, respectively [36]. According to the FMD vaccine evaluation criteria in Korea, it is known that host defense is possible when the VN titer induced by commercial vaccination is >1.65 (Log_10_).

Based on these criteria, the reduction period up to 1.65 (Log_10_), the upper limit of defense, and the reduction period down to 1.2 (Log_10_), the lower limit of defense, were very short for O1 Campos. For A2001 Argentina and A24 Cruzeiro, the antibody titers decreased much faster than those determined via SP A ELISA. Therefore, the defense conferred by MDAs, especially by VN titers, in piglets is considered very short, lasting about 4 weeks. The actual defense gap is expected to be longer due to the length of the antibody induction period after vaccination from the point of MDA decrease.

Compared to A2001 Argentina, the VN titer of A24 Cruzeiro was lower in sows but rather higher in piglets, and the overall half-life of VN titers was similar in A2001 Argentina and A24 Cruzeiro. The reasons for this phenomenon may be due to the following. (1) It is possible that the A24 Cruzeiro neutralizing antibody was transmitted at higher concentrations to piglets through the placenta during pregnancy. It is also likely that the VN titers for A24 Cruzeiro accumulated in piglets via the placenta were higher in fetuses than in sows just prior to parturition. Because oxygen, nutrients, antibodies to certain pathogens, and other immune response mediators are transmitted from mother to fetus through the placenta during pregnancy, these components may have been depleted in the mother before farrowing. (2) Newborns rely on an innate immune response to fight pathogens before the formation of active immunity. It is likely that postnatal exposure to various pathogens, along with environmental factors, temporarily elevated MDAs by activating the innate immune response and cooperating with the transmission of MDAs through breastfeeding to defend the host. (3) When breastfeeding after birth, some piglets may have consumed more sow’s milk. (4) Another possibility could be due to the intrinsic antigenicity (antigenic properties) or immunogenicity of the A24 Cruzeiro antigen. Although MDA delivered via placenta or breast milk also contained O1 Campos or A2001 Argentina-specific neutralizing antibody, the reason that A24 Cruzeiro-specific neutralizing antibody was specifically high in some piglets is as follows. Unlike other FMDV strains, due to the antigenicity (antigenic properties) or immunogenicity of A24 Cruzeiro contained in the vaccine, it is possible that it was accumulated at a higher concentration in the fetus or transmitted through breast milk intake.

However, it was difficult to obtain and study these commercial vaccine strains, and it was difficult to draw experimental evidence or conclusive interpretations about them in this study.

In addition to the FMD vaccines, pigs in South Korea are also vaccinated against swine fever, porcine reproductive and respiratory syndrome, porcine parvovirus, Aujeszky’s disease, porcine rotaviruses, and porcine epidemic diarrhea. Thus, changes in the concentration of total IgG, IgM, and IgA were determined and were similar to the patterns of change in antibody titers determined via SP O ELISA and SP A ELISA. Both the FMD vaccine-specific and non-specific antibodies were reduced in piglets throughout the early days after birth (Figure 5). Koch et al. reported that maternal IgG and IgA antibodies attenuated the early-life mucosal T helper cell response, and the absence of MDA induced a compensatory T cell-dependent immune response [37]. In addition, the regulatory T cell epitope of maternal IgG was found to activate a subset of natural regulatory T cells (nT_regs_), thereby inducing tolerance rather than immunogenicity [38,39]. Therefore, we determined that IgG, IgM, and IgA derived from sows could be delivered to piglets to stimulate T_regs_ and induce immune tolerance.

T_regs_ are a key subtype of T lymphocytes, which play an important role in regulating immune responses and maintaining immune tolerance under normal physiology as well as disease. Owing to their immunosuppressive properties, T_regs_ play a fundamental role in establishing the fetal–maternal immune tolerance necessary for successful pregnancy. T_regs_ cells play a major role in regulating exuberant immune responses and are suggested to dampen the protective immunity induced by infection and vaccination [19,40]. Furthermore, T_regs_ can suppress the proliferation and activation of a multitude of immune cell types. They employ a variety of mechanisms to mediate this suppression, and are thought to be flexible in this respect by adapting their mechanism according to their local environment. Ndure and Flanagan [41] proposed the strategy of targeting T_regs_ cells to improve vaccine immunogenicity in early life. In human studies, newborns acquire IgG antibodies from their mothers through the placenta, providing protection against infections that occur early in life. They described animal and human studies in which T_regs_ have been depleted in order to enhance vaccine responses. Finally, they suggested that a deeper understanding of the role that T_regs_ play in limiting or controlling vaccine-induced immunity would provide strategies to improve vaccine immunogenicity in a critical age group. Lin et al. [42] reported that vaccine-mediated antigen-specific T_regs_ attenuate antiviral immunity against acute influenza virus infection. They strategically inhibited T_reg_ development and induce a protective effect against heterogeneous influenza through vaccination capable of enhancing T cell immunity.

MDA levels decrease during the first 6 months of life and have usually disappeared by the end of the first year in humans. Several human studies have shown that MDAs inhibit humoral immune responses to infant vaccines. Human neonatal antibody responses are delayed in onset, are of shorter duration, achieve lower peak levels, and have lower affinity than adults [19,43,44]. To date, there have been no reports of the mention of T_regs_ in FMD vaccine studies. In this study, we aimed to determine the relationship between MDAs and T_regs_ transferred from sows vaccinated with FMD vaccine to piglets.

The expansion of T_regs_ decreases immune effector cells and increases the ratio and levels of suppressive cytokines such as IL-10 and TGFβ. The enhanced local and systemic inhibitory function of T_regs_ suppresses the T cell-mediated immune response by keeping DCs immature [45].

We verified the levels of T_regs_-related cytokines IL-2 [46], IL-10 [47], TGFβ [48] as well as IL-6, a Th2-related cytokine, and IL-12p40, which is mainly expressed in APCs, including DCs [49] (Figure 6), in order to determine whether MDA-induced interference was caused by T_regs._ Their reduction kinetics in sows and piglets were similar to those determined via SP ELISA, with rapid decreases at 7 to 14 dpb in sows after initially high levels of cytokine expression. In particular, IL-2, IL-10, and TGFβ showed a strong correlation with the age-dependent dynamics of MDAs, suggesting that in the presence of MDA, TCR-mediated B cell activation was likely to be inhibited by T_regs_. Recently, as IL-10 was shown to induce immunosuppression-related lymphophenia in mice with acute FMD infection, blocking IL-10/IL-10R signaling was suggested as a new therapeutic approach [50]. In contrast to the possibility that MDAs may temporarily exhibit protective effects in young individuals, IL-10 promotes lymphophenia, potentially causing severe clinical symptoms. Therefore, we suggest that MDA-induced interference is induced by the overexpression of T_reg_ cytokines. The expression levels of IL-2, IL-10, and TGFβ were 13.36, 47.22, and 45.62 pg/mL at 4 weeks of age (28 days of age), respectively. At 8 weeks (56 days) and 12 weeks (112 days), when piglets were vaccinated, the values were 5.88–3.68, 35.62–22.79, and 36.24–24.45 pg/mL, respectively. The time taken for VN titers to decrease to 1.2 Log_10_ was 8.75 days for O1 Campos, 21.51 days for A2001 Argentina, and 25.78 days for A24 Cruzeiro. Although the differences between FMDV type O and FMDV type A were significant, it is speculated that an effective immune response could be induced through vaccination in piglets when the IL-10 and TGFβ levels are below a specific threshold.

This study is the first report to elucidate the correlation between FMD vaccine-mediated MDA interference and T_reg_ response. However, verifying the effects of normalizing or suppressing T_reg_ on active immunity induction and finding the numerical threshold related T_regs_ that can overcome MDAs in newborn animal should be addressed in further studies.

Meanwhile, due to the limited supply of experimental animals, we only studied sows vaccinated with commercial vaccines from Biogénesis Bagó, which are widely used in South Korea, as well as the piglets from these sows. The MDA-related results may differ between individuals vaccinated using vaccines containing antigens other than O1 Campos, A2001 Argentina, and A24 Cruzeiro. In the future, additional studies on the characterization of MDAs induced by various vaccines should be carried out.

## 5. Conclusions

In conclusion, our findings will help determine the optimal timing of vaccination for overcoming MDAs and inducing a robust vaccine-mediated immune response in young individuals vaccinated against FMD disease. Further, they add to the understanding of MDA characteristics and interference in the host, which will contribute to establishing the strategy of a novel FMD vaccine development for overcoming MDA interference.

## Data Availability

Not applicable.

## Supplementary Materials

The following supporting information can be downloaded at: https://www.mdpi.com/article/10.3390/vaccines10050677/s1, Table S1: Abbreviation list.
