# Supplementary material for: Age-Dependent Dynamics of Maternally Derived Antibodies (MDAs) and Understanding MDA-Mediated Immune Tolerance in Foot-and-Mouth Disease-Vaccinated Pigs
Source: Vaccines (Basel). 2022 Apr 24;10(5):677. doi: 10.3390/vaccines10050677 (PMC9143745; doi:10.3390/vaccines10050677)
Supplement: Supplementary file 1 [file vaccines-10-00677-s001.zip › vaccines-1603879-supplementary.pdf]

Table S1. Abbreviation list

Abbreviation	Description
APCs	antigen presenting cells
DCs	dendritic cells
dpb	days post birth
FcRn	neonatal Fc receptor
FMD	foot-and-mouth disease
FMDV	foot-and-mouth disease virus
MDA	maternally-derived antibodies
NK	natural killer
NK T	natural killer T
OIE	World Organization for Animal Health
PD_{50}	50% protective dose
PI	percent inhibition
TCID	tissue culture infectious dose
T_{eff}	effector T cells
T_{regs}	regulatory T cells
SP	structural protein
VN	virus-neutralizing
